# A highly glucose-tolerant GH1 β-glucosidase with greater conversion rate of soybean isoflavones in monogastric animals

**DOI:** 10.1007/s10295-018-2040-6

**Published:** 2018-05-09

**Authors:** Huifang Cao, Yueqi Zhang, Pengjun Shi, Rui Ma, Hong Yang, Wei Xia, Ying Cui, Huiying Luo, Yingguo Bai, Bin Yao

**Affiliations:** 1 Key Laboratory for Feed Biotechnology of the Ministry of Agriculture, Feed Research Institute, Chinese Academy of Agricultural Sciences, No. 12 Zhongguancun South Street, Beijing, 100081 People’s Republic of China; 2 College of Animal Science, Zhejiang University, Hangzhou, 310058 People’s Republic of China

**Keywords:** Glycoside hydrolase (GH) family 1, β-Glucosidase, Glucose tolerance, Soybean isoflavones, Feed additive

## Abstract

In the feed industry, β-glucosidase has been widely used in the conversion of inactive and bounded soybean isoflavones into active aglycones. However, the conversion is frequently inhibited by the high concentration of intestinal glucose in monogastric animals. In this study, a GH1 β-glucosidase (*As*BG1) with high specific activity, thermostability and glucose tolerance (IC_*50*_ = 800 mM) was identified. It showed great glucose tolerance against substrates with hydrophobic aryl ligands (such as *p*NPG and soy isoflavones). Using soybean meal as the substrate, *As*BG1 exhibited higher hydrolysis efficiency than the GH3 counterpart Bgl3A with or without the presence of glucose in the reaction system. Furthermore, it is the first time to find that the endogenous β-glucosidase of soybean meal, mostly belonging to GH3, plays a role in the hydrolysis of soybean isoflavones and is highly sensitive to glucose. These findings lead to a conclusion that the GH1 rather than GH3 β-glucosidase has prosperous application advantages in the conversion of soybean isoflavones in the feed industry.

**Electronic supplementary material:**

The online version of this article (10.1007/s10295-018-2040-6) contains supplementary material, which is available to authorized users.

## Introduction

Soybean isoflavone, having a structure similar to estrogen, is approximately 1.2–4.2 g/kg in soybean meal feed [20]. There are three free isoflavone aglycones (daidzein, genistein and glycitein) and nine bounded isoflavone glycosides, and those bounded ones account for 95–97% of soybean isoflavone. The isoflavone aglycones have attracted considerable attention for their distinguished functions in promoting animal growth, improving feed utilization, improving reproductive ability, etc. [10, 14]. Thus, how to transform the bounded glycosides into free forms in the intestinal tract of monogastric animals is of economic and environmental importance [13, 26].

β-Glucosidase is a glycoside hydrolases (GH) that acts on the glycosidic bonds of carbohydrate moiety to release nonreducing terminal glycosyl residues, glycoside, and oligosaccharides [2, 3]. Based on the sequences of catalytic domains, β-glucosidases are grouped into GH 1, 3, 5, 9, 30 and 116 (http://www.cazy.org/) [4, 11], and most of them are confined in families 1 and 3. Based on the substrate specificity, β-glucosidases are grouped into aryl-β-glucosidase, true cellobiase, and β-glucosidase with broad substrate specificity [24, 30]. Those β-glucosidases acting on cellobiose and soybean isoflavones have important application values in the feed, food, biofuel and other fields [25, 32, 34]. One bottleneck of β-glucosidase application in the feed industry is its sensitivity to glucose, which is commonly assessed by measuring the half inhibitory concentration of glucose (IC_*50*_) or inhibition constant (*K*_*i*_). Recent studies have shown that GH1 and GH3 β-glucosidases vary in their tolerance to glucose. In comparison to the GH3 β-glucosidases that have IC_*50*_ values of less than 100 mM [6, 16, 36] due to the competitive inhibition by glucose [5, 12, 31], the GH1 counterparts have an IC_*50*_ value of several hundreds to thousands of millimole [28, 33, 35]. A few studies have been carried out to reveal the high glucose tolerance mechanism of GH1 β-glucosidases. Giuseppe et al. compared the catalytic pocket structures of GH1 and GH3 β-glucosidases and found that the deeper catalytic pocket of GH1 β-glucosidases may account for their high glucose tolerance [9]; Yang et al. ascribed the high glucose tolerance of GH1 β-glucosidases to the substrate binding to multiple non-catalytic sites and their transglycosylation activities [40]. These studies gave some hints on the glucose tolerance mechanism of GH1 β-glucosidases [41], but no general accepted view was proposed.

In the intestinal tract of monogastric animals, the conversion efficiency of soybean isoflavones is low mainly due to the deficiency of β-glucosidase and the competitive inhibition of glucose [38]. One practice is to supplement exogenous β-glucosidase into soybean meal feed to increase the utilization of soy isoflavones [21, 29], the other is to screen and engineer the β-glucosidase with high glucose tolerance. The present study aims to obtain a β-glucosidase with great glucose tolerance and conversion efficiency of soybean isoflavones for potential application in the feed industry. A GH1 β-glucosidase (*As*BG1) with high specific activity, thermostability and glucose tolerance (IC_*50*_= 800 mM) was identified from *Alicyclobacillus* sp. A4. Biochemical characterization showed that *As*BG1 had glucose tolerance only against substrates with hydrophobic aryl ligands (such as *p*NPG and soy isoflavones). Further comparison of the hydrolysis capabilities of GH1 and GH3 β-glucosidases on soy isoflavones revealed that GH1 β-glucosidases have more application advantages and more prosperous potentials than GH3 ones in the feed industry.

## Materials and methods

### Strains, culture media, and reagents

The acidothermophilic *Alicyclobacillus* sp. A4 (whole genome sequenced) isolated from the hot spring water was stored in our laboratory [1]. GH3 β-glucosidase Bgl3A derived from *Talaromyces leycettanus* JCM12802 [376] was used as the reference. Ni^2+^-affinity beads from Suzhou Beaver Biomedical Engineering (China), and glucose oxidation (GOD-POD) kit from Beijing Leadman (China) were purchased. Substrates of *p*-nitrophenyl β-d-glucopyranoside (*p*NPG), *p*-nitrophenyl α-l-arabinofuranoside (*p*NPAf), *p*-nitrophenyl β-d-xylopyranoside (*p*NPX), barley β-glucan, lichenan, Avicel, and standard samples of daidzein, glycitein and genistein from Sigma-Aldrich (USA), cellobiose to cellohexose, laminaritetraose and maltose from Megazyme (Ireland), and daidzin from Tokyo Chemical Industry were obtained. The soybean meal was purchased from local market. All the other reagents were of analytical grade and commercially available.

### Gene cloning and sequence analysis

Based on the genomic annotation of *Alicyclobacillus* sp. A4, a β-glucosidase gene of GH1, *Asbg1*, was identified. To obtain the full-length coding gene, a specific primer set *Asbg1*-F and *Asbg1*-R harboring 3 protection nucleotides, the restriction site of *Eco*RI or *Hind*III and 22 nucleotides of the 5′- and 3′-ends of *Asbg1* (Table 1) were designed for the PCR amplification. The PCR products were purified, linked with vector pEASY-T3, and transformed into *E. coli* Trans1-T1 for sequencing.

**Table 1:** Primers used in this study

Name	Sequence (5′ → 3′)	Size (bp)
*Asbg1*-F	ATGAGTCAAAACCTTTCGTTTCCGG	25
*Asbg1*-R	TTATACCGTTCCTTGCACGGTTTCT	25
*Asbg1*-pET30a-F	tttaagaaggagatatacatatgAGTCAAAACCTTTCGTTTCCGGACGAT	50
*Asbg1*-pET30a-R	cagtggtggtggtggtggtgTACCGTTCTTGCACGGTTTCTGGCCAAGCT	50
pET30a-*Asbg1*-F	AAACCGTGCAAGGAACGGTAcaccaccaccaccaccactgagatccggct	50
pET30a-*Asbg1*-R	AACGAAAGGTTTTGACTcatatgtatatctccttcttaaagttaaacaaa	50

### Expression and purification

To produce the gene product in *E. coli*, four specific primers, *Asbg1*-pET30a-F and *Asbg1*-pET30a-R (each contained 20 bp of the pET30a sequence and 30 bp of the *Asbgl* sequence), and pET30a-*Asbg1*-F and pET30a-*Asbg1*-R (each contained 20 bp of the *Asbgl* sequence and 30 bp of the pET30a sequence) were designed (Table 1) to construct the recombinant expression plasmid pET30a-*Asbg1*. The target gene *Asbg1* was ligated to the expression vector pET30a by overlap PCR method. The positive clones were verified by DNA sequencing, and then transformed into the *E. coli* BL21 (DE3) competent cells. The expression of recombinant *A*sBG1 was induced by isopropyl-β-d-thiogalactopyranoside (IPTG), and the crude enzyme was purified by Ni^2+^-affinity beads.

### Assay of the enzymatic activity

One unit of enzymatic activity (U) was defined as the amount of β-glucosidase required to hydrolyze the substrate to produce 1 μmol of product per minute under certain reaction conditions. Each assay had triplicates. The assay methods for different substrates were described as below:

A. *p*NP glycoside substrates. The reaction systems containing 250 μL of 4 mM *p*NP glycoside substrates and 250 μL of appropriately diluted enzyme solution were incubated at 55 °C and pH 6.5 for 10 min, followed by the addition of 1.5 mL of 1 M Na_2_CO_3_ to terminate the reaction. After cooling down to room temperature, the absorbance at OD_405_ was measured. The enzymatic activity was determined based on the amount of *p*NP released.
B. Reducing oligosaccharide substrates. The GOD-POD method [22] was used. Reaction systems containing 70 μL of 4 mM oligosaccharide and 70 μL of appropriately diluted enzyme solution were incubated at 55 °C and pH 6.5 for 10 min, followed by 5 min-boiling water bath to terminate the reaction. After addition of 2.1 mL GOD reagent in the glucose oxidation kit, the reaction systems were incubated at 37 °C for 10 min. After cooling down to room temperature, the absorbance at OD_520_ was measured. The enzymatic activity was determined based on the amount of glucose released.
C. Polysaccharide substrates. The DNS method [23] was used. The substrate solution, 900 μL of 0.5% (w/v) barley β-glucan, CMC-Na or lichenan, was preheated in a water bath for 2 min at 55 °C, followed by the addition of 100 μL of appropriately diluted enzyme solution. After incubation at 55 °C and pH 6.5 for 10 min, the reactions were terminated by adding 1.5 mL of DNS and boiling for 5 min. The OD_540_ values were then measured. The enzymatic activity was determined based on the amount of reducing sugar released.

### Biochemical characterization

*p*NPG was used as the substrate for biochemical characterization of purified recombinant *As*BG1. The optimal pH for *As*BG1 was determined at 55 °C for 10 min in the buffers with pH 3.0–10.0. The optimal temperature was examined at the optimal pH 6.5 under the temperature range of 30‒80 °C. The pH stability was examined by measuring the residual enzyme activity under the standard conditions (pH 6.5 and 55 °C for 10 min) after pre-incubation of the enzyme without substrate in various pH buffer mentioned above for 1 h at 37 °C. And the thermostability was determined by measuring the residual activity under the standard conditions after the pre-incubation at 50 and 60 °C for 0, 5, 10, 20, 30, and 60 min without the substrate, respectively.

### Substrate specificity and kinetic parameters

To scan the substrate spectrum, 2 mM of *p*NP materials (*p*NPG, *p*NPX and *p*NPAf), 5 mM of oligosaccharides (cellobiose to cellohexaose, laminaritetraose, and maltose), and 0.5‒1% (w/v) polysaccharides (lichenan, barley β-glucan, and Avicel) were used as substrates for *As*BG1 activity assays under standard conditions (pH 6.5 and 55 °C for 10 min).

The initial reaction rate, *V* (μmol min mg^−1^), was determined under optimal conditions (pH 6.5 and 55 °C for 5 min) with 0.2‒1.5 mM *p*NPG or 1‒8 mM cellobiose as the substrate. A Lineweaver–Burk plot was drawn with the reciprocals of the substrate concentration (1/s, mM^−1^) and reaction rate (1/*V*, μmol^−1^ min^−1^ mg) as the *X*- and *Y*-axis, respectively. The substrate affinity, *K*_m_ (mM) and the reaction velocity maximum, *V*_max_ (μmol min mg^−1^), were calculated according to the formula 1/*V* = *K*_m_/*V*_max_ × (1/s) + 1/*V*_max_, and the catalytic efficiency constant *k*_cat_/*K*_m_ (s^−1^ mM^−1^) = *V*_max_× MW/60/*K*_m_, where MW represents the relative molecular mass (kDa) of *As*BG1.

### *As*BG1 tolerance to monosaccharides and *p*NP

With *p*NPG as the substrate, the *As*BG1 tolerance to different monosaccharides was determined. Reaction systems containing 125 μL of 8 mM *p*NPG, 250 μL of 0‒3 M monosaccharides (glucose, xylose, arabinose, galactose, fructose, and mannose), and 125 μL of appropriately diluted enzyme were incubated at 55 °C and pH 6.5 for 10 min, followed by the addition of 1.5 mL of 1 M Na_2_CO_3_ to terminate the reaction. The absorbance at OD_405_ was measured.

Glucose tolerance of *As*BG1 with cellobiose as the substrate was also assessed. The reaction systems containing 100 μL of 8 mM cellobiose, 100 μL of glucose solution (at the final different concentration of 0, 40 or 80 mM) and 200 μL of the appropriately diluted enzyme solution were incubated at 55 °C and pH 6.5 for 10 min, followed by 5 min-boiling water bath. After cooling down to room temperature, the hydrolysis products were collected through a Vivaflow ultrafiltration membrane (Vivascience, Germany) with a molecular weight cut-off of 5 kDa, diluted 1000-fold with ddH_2_O, and analyzed by high performance ion exchange chromatography (HPIEC) with 1 μg mL^−1^ glucose as the standard. The substrate hydrolysis rate of *As*BG1 was determined as follows: hydrolysis rate (%) = 100% × (*A*_c_ − *A*_t_)/*A*_c_, where *A*_c_ and *A*_t_ represent the substrate amounts determined in the blank (without enzyme addition) and treatment (with enzyme addition) samples, respectively.

The *As*BG1 tolerance to *p*NP with *p*NPG and cellobiose as substrates was also assessed. The reaction systems consisted of 2 mM of *p*NPG or cellobiose, different concentrations of *p*NP (at the final concentration of 0, 10, 20, and 30 mM) and appropriately diluted enzyme solution.

### Glucose tolerance against daidzin hydrolysis

Purified *As*BG1 (GH1 family) is obtained from this study with the optimum pH 6.5 and Bgl3A (GH3 family) is available from our lab with the optimum pH 4.5 [37]. In this study, *As*BG1 and Bgl3A were used as the comparative materials to hydrolysis daidzin. Reaction system containing 100 μL of 2 mM daidzin, 100 μL of 80 mM glucose and 200 μL of enzyme solution (0.036 U *As*BG1 or Bgl3A) were incubated under the simulated intestinal conditions (37 °C and pH 6.5) for 5 min and terminated by a boiling water bath for 5 min. After cooling down to room temperature, the supernatants were collected by centrifugation at 12,000 rpm for 10 min, and then analyzed by HP1100 HPLC system (Waters, USA) equipped with a Diamonsil C18 column (5 μm × 250 mm × 4.6 mm; Dima, USA) with 2 mM daidzin as the standard. Reactions without enzyme or glucose addition were treated as controls. The conversion efficiency was determined based on the reduced amount of daidzin. Hydrolysis rate (%) = 100% × (*B*_c_ − *B*_t_)/*B*_c_, where *B*_c_ and *B*_t_ represent the amounts of daidzin in the control and treatment samples.

### Conversion of soybean isoflavones under simulated intestinal conditions

Simulated soybean isoflavone hydrolysis by β-glucosidase *As*BG1 or Bgl3A in the intestinal tract of monogastric animals was conducted with soybean meal as the substrate. Reaction systems containing 5 g crushed and sieved soybean meal and 15 mL buffer (containing 10 U enzyme and/or 10 mM glucose) were incubated at 37 °C at pH 6.5 for *As*BG1 or pH 4.5 for Bgl3A in a water bath shaker for 2 h, followed by the addition of 35 mL of absolute ethanol and extraction of production at 65 °C for 2 h. The supernatants were then collected by high-speed centrifugation for HPLC analysis. Daidzein, glycitein and genistein were used as the standard samples for the quantitative analysis.

## Results

### Gene cloning and sequence analysis

The GH1 β-glucosidase-encoding gene, *Asbg1* (GenBank accession no. KY039184), was obtained from the genome of *Alicyclobacillus* sp. A4. The full-length gene contained 1365 bp, and coded for 454 amino acid residues. Sequence analysis indicated that deduced *As*BG1 had no putative signal peptide sequence and had a calculated molecular mass of 51.7 kDa. Multi-sequence alignment showed that *As*BG1 had E166 and E354 as the catalytic residues.

### Protein expression and purification

**Fig. 1:**
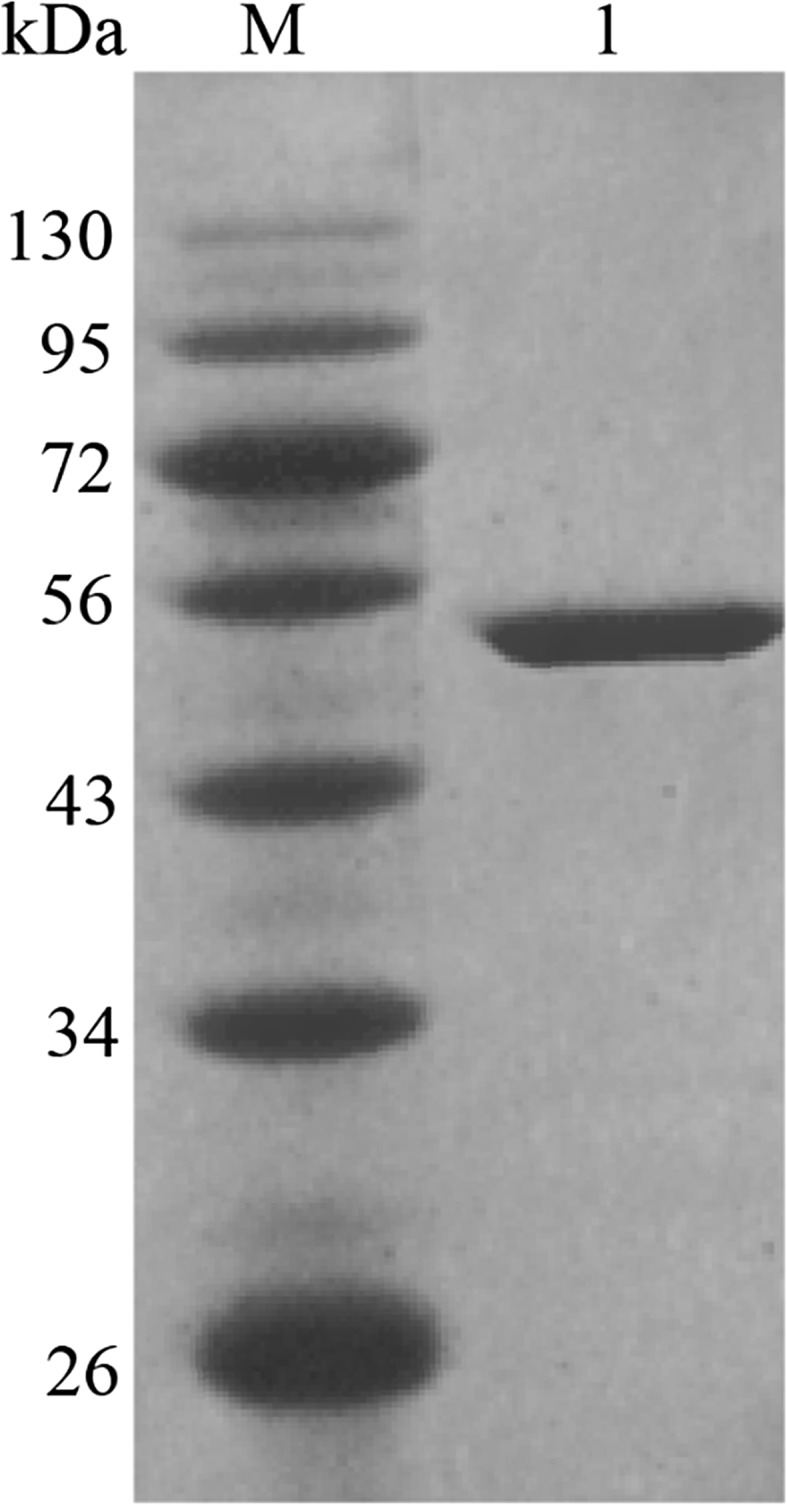
SDS-PAGE analysis of the purified recombinant *As*BG1. Lane M, the standard protein molecular weight markers; and lane 1, the purified recombinant *As*BG1

The recombinant plasmid pET30-*Asbg1* was successfully constructed and transformed into *E. coli* BL21(DE3) competent cells for production. After IPTG induction and cell sonication, the cell lysate showed a β-glucosidase activity of approximately 100 U/mL. The crude enzyme was purified to homogeneity by Ni^+^ affinity beads. Purified recombinant *As*BG1 migrated a single band of approximately 52 kDa in SDS-PAGE, which was consistent with the theoretical molecular weight (Fig. 1).

### Biochemical characterization

**Fig. 2:**
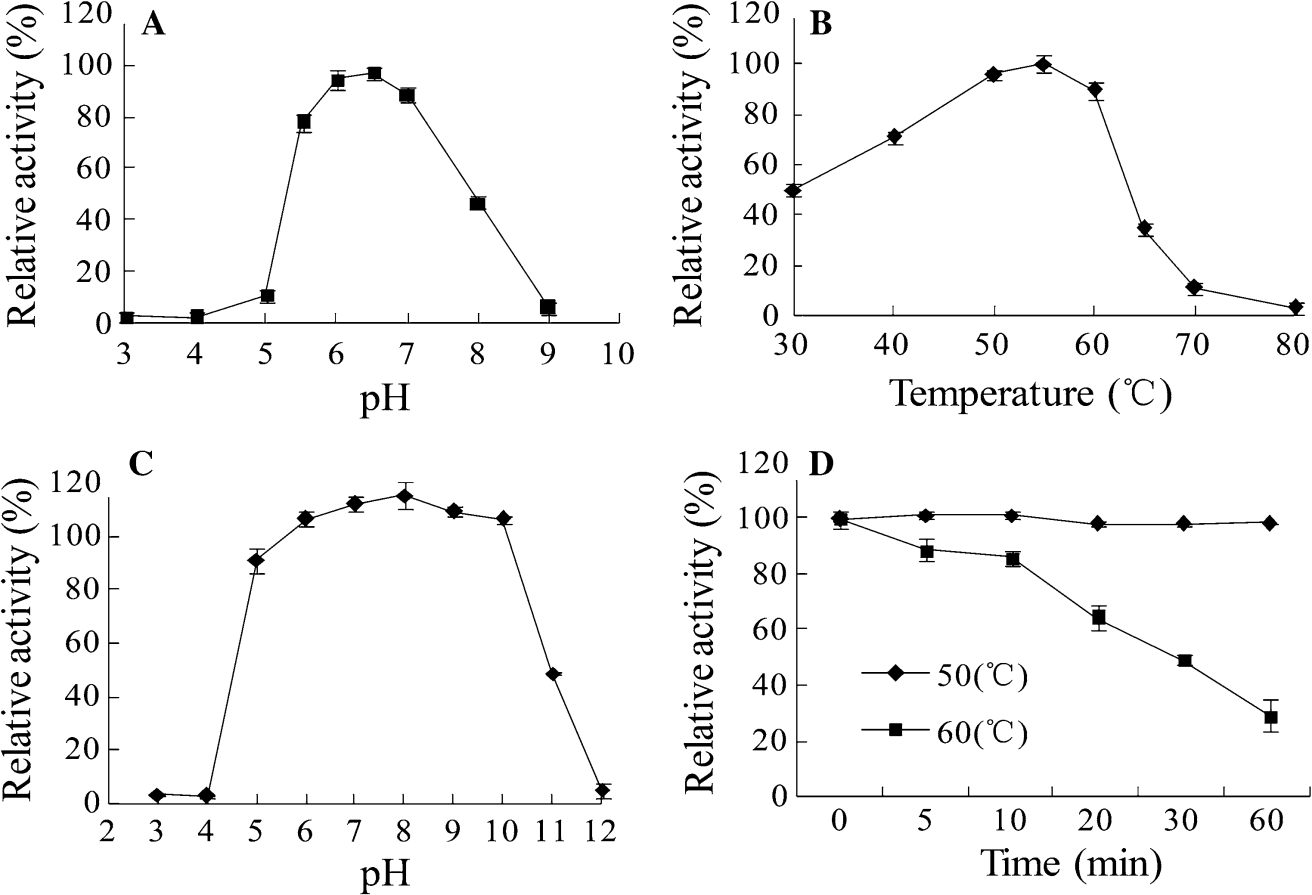
Characterization of the purified recombinant *As*BG1. **a** Effect of pH on the *As*BG1 activity. **b** Effect of temperature on the *As*BG1 activity. **c** pH stability. **d** Thermostability assay. Each value in the panel represents the mean ± SD (*n* = 3)

Using *p*PNG as the substrate, purified recombinant *As*BG1 displayed optimal activity at pH 6.5 and remained 70% of the maximal activity at pH 5.5–7.5 (Fig. 2a). Under the optimal pH, the enzyme exhibited the maximal activity at 55 °C, and remained highly active over the temperature range of 30‒60 °C (Fig. 2b). *As*BG1 was stable over the acidic–alkaline range (pH 5.0–10.0), retaining more than 80% of the initial activity after 1-h incubation at 37 °C without substrate (Fig. 2c). The thermostability of *As*BG1 was assayed. It retained highly stable after 1-h incubation at 50 °C, but lost up to 80% activity at 60 °C under same conditions (Fig. 2d).

### Substrate specificity and kinetic parameters

The *As*BG1 had a broad substrate spectrum including oligosaccharides of both β-(1,4)- and β-(1,3)-glycosidic linkages (Table 2) of the substrates tested, *As*BG1 exhibited the highest activities against *p*NPG, cellooligosaccharides and laminaritetraose. The *As*BG1 activities towards cellotriose, cellotetraose, cellopentose and cellohexaose were significantly higher, approximately twofold, than that to cellobiose. Besides, it had weak or detectable activities towards *p*NPX, *p*NPAf, maltose, and polysaccharides barley β-glucan, Avicel, and lichenan.

**Table 2:** Substrate specificity of the *As*BG1

Substrates	Glycosyl linkage	Concentration	Specific activity (U/mg)^a^
*p*NPG	β-glucose	2 mM	50 ± 0.2
*p*NPX	β-xylose	2 mM	1.1 ± 0.0
*p*NPAf	β-arabinofuranoside	2 mM	0.5 ± 0.0
Cellobiose	4-*O*-β-d-Glc-d-Glc	5 mM	21 ± 0.1
Cellotriose	[β-d-Glc-1,4)]_2_-d-Glc	5 mM	39 ± 0.0
Cellotetraose	[β-d-Glc-1,4)]_3_-d-Glc	5 mM	40 ± 1.1
Cellopentaose	[β-d-Glc-1,4)]_4_-d-Glc	5 mM	42 ± 1.4
Cellohexaose	[β-d-Glc-1,4)]_5_-d-Glc	5 mM	42 ± 2.8
Laminaritetraose	[β-d-Glc-1,3)]_3_-d-Glc	5 mM	35 ± 1.6
Maltose	4-*O*-a-d-Glc-d-Glc	5 mM	0.5 ± 0.0
Barley β-glucan	1,3:1,4-β-d-glucan	0.5%	7.5 ± 0.1
Lichenan	1,3:(1,4)_2_-β-d-glucan	0.5%	2.5 ± 0.0
Avicel	1,4-β-d-glucan	1.0%	1.2 ± 0.0

Each value in the panel represents the mean ± SD (*n* = 3)

^a^The enzymatic activity towards 2 mM *p*NPG or 5 mM cellobiose was set to be 100%. Values are shown as the mean ± SD (*n* = 3)

When using *p*NPG as the substrate, the purified recombinant *As*BG1 had the *K*_m_, *V*_max_ and *k*_cat_/*K*_m_ values of 0.4 mM, 111 μmol min^−1^ mg^−1^, and 215 s^−1^ mM^−1^, respectively. The *K*_m_, *V*_max_ and *k*_cat_/*K*_m_ values of *As*BG1 with cellobiose were 7.3 mM, 54 μmol min^−1^ mg^−1^, and 9 s^−1^ mM^−1^, respectively.

### *As*BG1 tolerance to monosaccharides and *p*NP

**Fig. 3:**
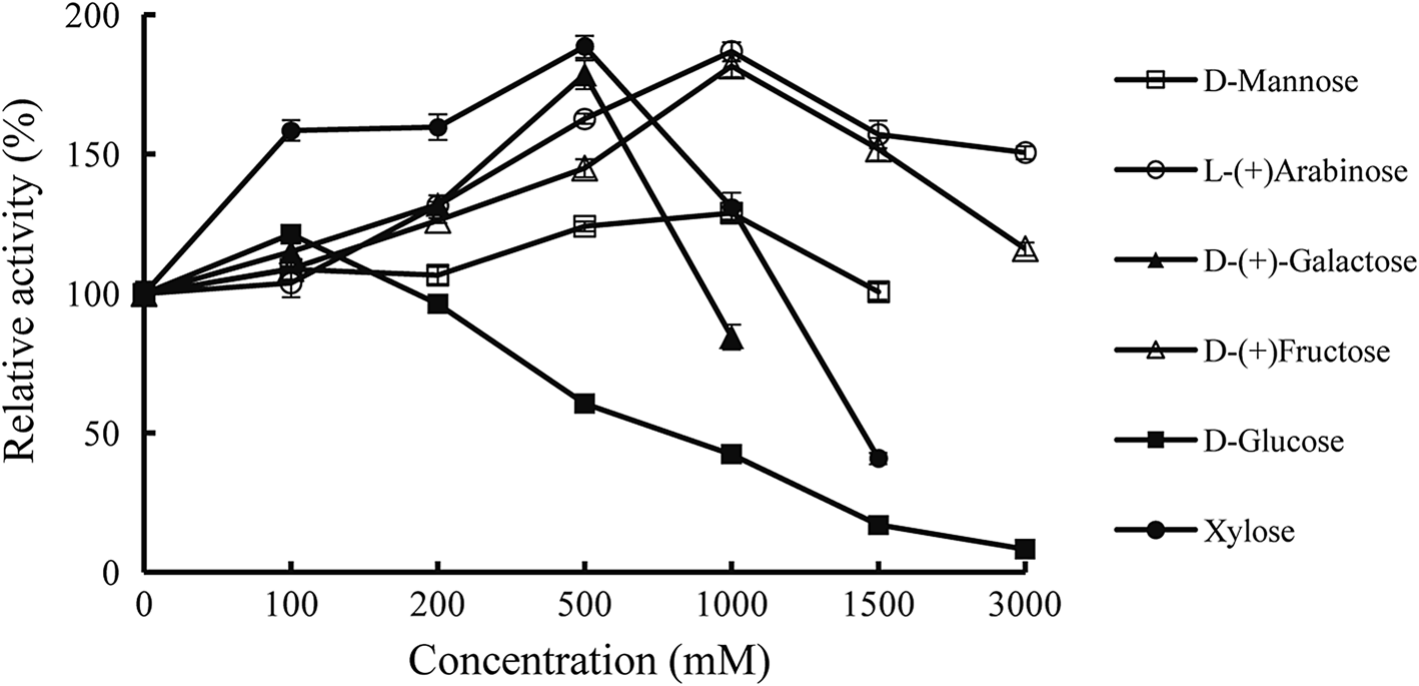
*As*BG1 tolerance to various monosaccharides. Each value in the panel represents the mean ± SD (*n* = 3)

When using *p*NPG as the substrate, *As*BG1 was highly tolerant to a variety of monosaccharides (Fig. 3). In the presence of 50‒200 mM glucose, *As*BG1 showed enhanced activities up to 120%. Other tested oligosaccharides (including galactose, xylose, arabinose, galactose and fructose) at the concentration of ≤ 500 mM stimulated the *As*BG1 activity by 1.2–1.8-fold. When increased the concentration to 3 M, arabinose and fructose still had stimulatory effects on *As*BG1 activity.

**Fig. 4:**
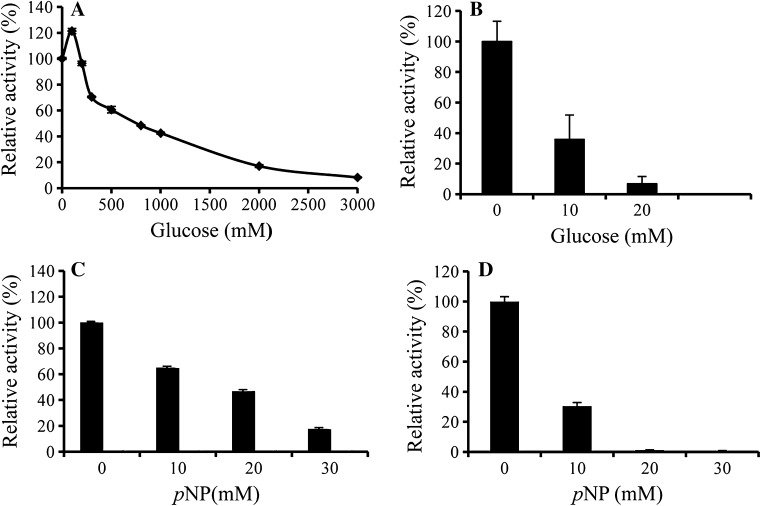
*As*BG1 tolerance to glucose and *p*NP. **a** Glucose tolerance in the presence of 2 mM *p*NPG; **b** Glucose tolerance in the presence of 2 mM cellobiose; **c** *p*NP tolerance in the presence of 2 mM *p*NPG; **d** *p*NP tolerance in the presence of 2 mM cellobiose. Controls without addition of glucose or *p*NP were defined as 100%. Each value in the panel represents the mean ± SD (*n* = 3)

The *As*BG1 tolerance to *p*NP and glucose with *p*NPG and cellobiose as the substrate was also tested. When using pNPG as the substrate, AsBG1 had an IC50 value of up to 800 mM glucose (Fig. 4a). With cellobiose as the substrate, 10 and 20 mM glucose inhibited approximately 60 and 90% of enzyme activities (Fig. 4b). The product *p*-nitrophenol (*p*NP) strongly inhibited the *As*BG1 activity. As shown in Fig. 4c, 10 mM *p*NP inhibited 40% of the enzyme activity. With cellobiose as the substrate, the presence of 10 mM *p*NP caused the 70% activity lost (Fig. 4d).

### Glucose tolerance against daidzin hydrolysis

Under simulated intestinal conditions (10 mM glucose, 0.5 mM daidzin, 37 °C, pH 6.5, and 5 min), the hydrolysis rates of *As*BG1 and Bgl3A, each at 0.036 U, were determined. Bgl3A converted 15 and 26% daidzin with and without glucose, while *As*BG1 degraded 21 and 20% daidzin under the same conditions. The results indicated that the conversion efficiency of daidzin by Bgl3A was seriously impeded by glucose. Instead, the presence of glucose even stimulated the enzymatic activity of *As*BG1 towards daidzin.

### Simulated conversion of soybean isoflavones in intestinal tract

**Fig. 5:**
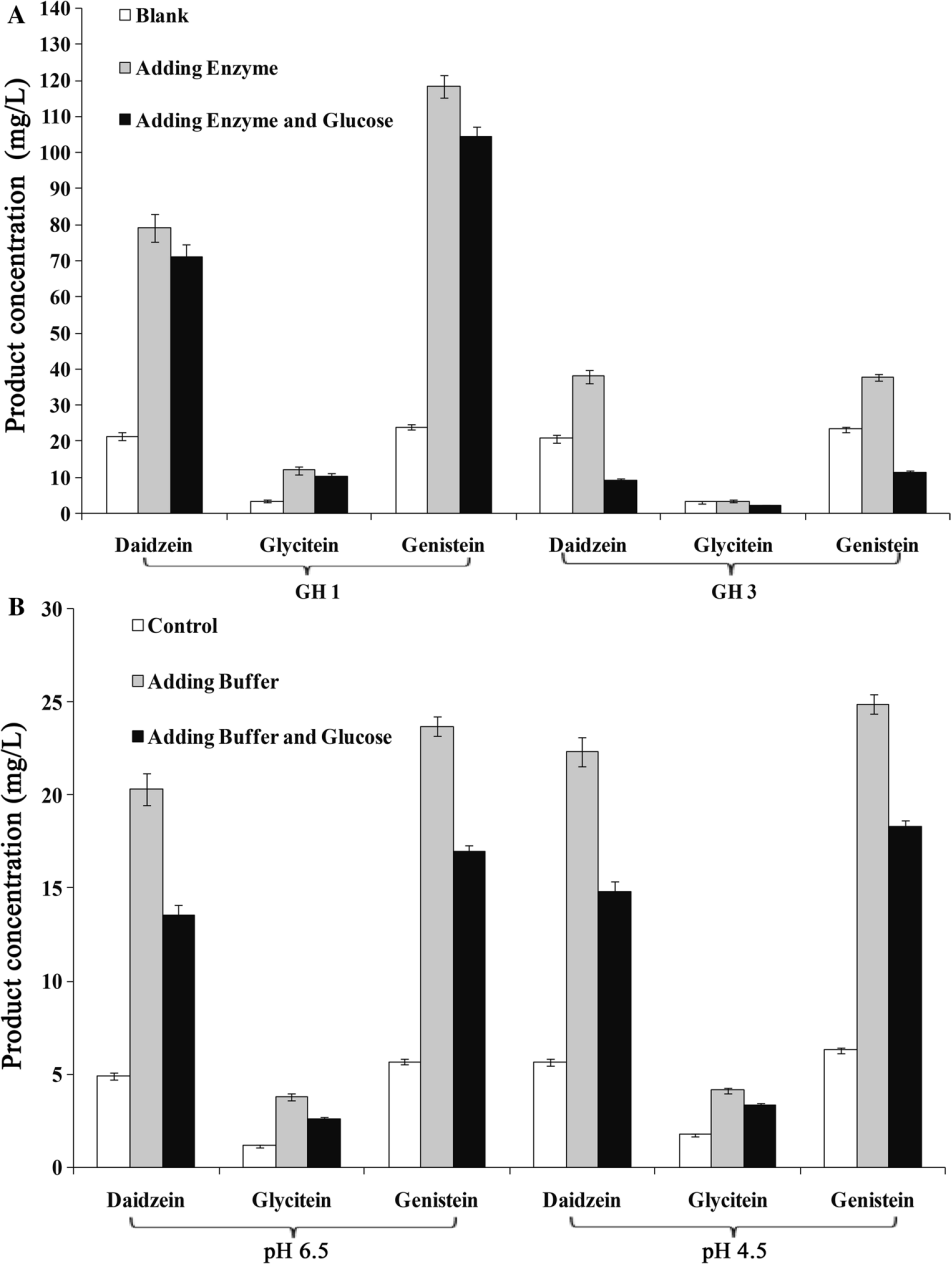
Hydrolysis efficiencies of *As*BG1 (GH1) and Bgl3A (GH3) on soy isoflavones in soybean meal. **a** Glucose influence on hydrolysis efficiency with exogenous enzyme **b** without exogenous enzyme. Each value in the panel represents the mean ± SD (*n* = 3)

The conversion efficiencies of *As*BG1 and Bgl3A towards soybean isoflavones were compared with and without glucose addition (Fig. 5a). Without enzyme addition, 22 mg L^−1^ daidzein, 4 mg L^−1^ glycitein and 24 mg L^−1^ genistein were detected in the reaction mixtures. When 10 U *As*BG1 were added, the amounts of daidzein, glycitein and genistein released with and without glucose were increased to 71–79 mg L^−1^, 10–12 mg L^−1^, 104–118 mg L^−1^, which had no statistical differences. In contrast, the amounts of daidzein (38 mg L^−1^), glycitein (4 mg L^−1^) and genistein (38 mg L^−1^) released by Bgl3A were much less than those of *As*BG1. The presence of 10 mM glucose further decreased the conversion efficiencies of Bgl3A by 9, 2, 12 mg L^−1^, and the ANOVA analysis showed significant differences between the two groups of with and without glucose addition of Bgl3A.

Based on the observed results above, the degradation of soybean meal by itself was further studied without adding exogenous enzyme solution (Fig. 5b). The results showed that the initial concentrations of daidzein, glycitein and genistein in the control group (reaction for 0 h) for both GH1 and GH3 were about 5, 2 and 6 mg L^−1^, respectively. After reaction time of 2 h, the production amount of daidzein, glycitein and genistein in the first reaction group (adding buffer only) for both GH1 and GH3 were increased to about 20, 4 and 24 mg L^−1^. After reaction time of 2 h, the production amount of daidzein in the second reaction group (adding buffer and 10 mM glucose) for both GH1 and GH3 was decreased about 33% lower than that of the first reaction group (adding buffer only).

## Discussion

The genus *Alicyclobacillus* is well-known for its capability of producing various hydrolases. In a previous study, a β-glucosidase of GH1, *Aa*β-gly (WP_008336965.1), has been identified in *A. acidocaldarius* and biochemically characterized [17]. *Asbg1* under this study is the second known GH1 β-glucosidase from *Alicyclobacillus*. These two β-glucosidases shared 69% amino acid sequence identity and were both heterologously produced in *E. coli*. In comparison to the recombinant *Aa*β-gly that has optimal activities at 65 °C, the purified recombinant *As*BG1 has a temperature optimum of 55 °C and remains more active over the moderate temperature range (30‒60 °C). Therefore, the *As*BG1 is more favorable for application in the food and feed industries.

*As*BG1 was distinguished for its broad substrate specificity. It acted on both oligosaccharides (cellooligosaccharides and laminarioligosaccharides) and polysaccharides (barley β-glucan, lichenan, and Avicel). Of the five cellooligosaccharides tested, *As*BG1 demonstrated much higher activities, approximately twofold, towards cellotriose to cellohexaose than to cellobiose. The results are contrary to the β-glucosidases derived from *Aspergillus oryzae* and *Aspergillus niger* with increased activities on cellobiose [27, 39]. The catalytic efficiencies of *As*BG1 towards *p*NPG and cellobiose were 215 and 9 s^−1^ mM^−1^, respectively, which are much higher than the β-glucosidases from *Humicola insolens* and *Trichoderma reesei* [18, 33], but lower than *Aa*β–gly [17]. The extensive substrate specificity and relatively high catalytic efficiency make *As*BG1 potentially applicable in the feed, bioenergy, food, textile and other fields.

A few studies have shown that the glucose tolerance of GH1 β-glucosidase is much stronger than that of GH3 counterparts [7, 8, 15, 19]. Although great efforts have been exerted to analyze the glucose tolerance mechanism of GH1 β-glucosidases using molecular mutation techniques and molecular dynamics simulations [5, 18, 33], no general accepted view has been proposed so far. In the present study, *As*BG1 showed great tolerance to a variety of monosaccharides (glucose, lactose, arabinose, galactose, fructose, and xylose) with *p*NPG as the substrate. Glucose as one of the most important product in many industrial processes showed stimulatory effects (up to 120%) on the *As*BG1 activity at the concentration of 200 mM, with the IC_*50*_ value of up to 800 mM. However, when using cellobiose as the substrate, the enzyme showed sensitivity to both glucose and *p*NP. These results indicated that the high glucose tolerance of *As*BG1 only functioned on aromatic substrate *p*NPG. The result of daidzin hydrolysis further demonstrated that the conversion efficiency of daidzin by Bgl3A (GH3) was seriously impeded by glucose while *As*BG1 was not. These findings indicated the greater potential advantage of GH1 than GH3 β-glucosidases in the feed industry.

Soybean isoflavone, as an important component of corn soybean meal feed, plays an important role in promoting animal growth and improving feed conversion rate. The performance of *As*BG1 hydrophobic aryl ligands and Bgl3A in the hydrolysis of soybean isoflavone in soybean meal was also compared. With three free, active aglycones (daidzein, glycitein, genistein) in soybean isoflavones as target products, the soybean meal was treated with or without GH1/3 β-glucosidase addition. The results showed that the hydrolysis capability of *As*BG1 on soybean isoflavone was much stronger than that of Bgl3A, with the higher daidzein yield of 52%. With the presence of 10 mM glucose, the daidzein yield by *As*BG1 hydrolysis reached 88% higher than that of Bgl3A. It is surprising to find that the daidzein yield of the reaction groups (with addition of enzyme and glucose) was 55% lower than that of the blank group (with buffer only). Further analysis revealed that the endogenous β-glucosidase contained in soybean meal might play a role in the hydrolysis. Sequence analysis on the endogenous β-glucosidase of soybean meal indicated that most of them belong to GH3, while those of GH1 frequently are mainly intracellular without signal peptide. These exogenous and endogenous β-glucosidases of GH3 might be inhibited by the glucose in the reaction system.

In summary, a neutral mesophilic β-glucosidase of GH1 with broad substrate specificity was identified in *Alicyclobacillus* sp. A4 and functionally verified in *E. coli* BL21. This study demonstrated, for the first time, that the GH1 β-glucosidase only showed glucose tolerance against substrates with hydrophobic aryl ligands (such as *p*NPG and soy isoflavones) but not substrates such as cellobiose. In the hydrolysis process of soybean isoflavone in soybean meal, GH1 exhibited better hydrolysis efficiency than GH3 β-glucosidase with or without glucose addition. Besides, it is also the first time to find that the endogenous β-glucosidase contained in soybean meal plays a role in the hydrolysis of soybean isoflavone to some extent and is strongly inhibited by glucose. All of these findings showed that GH1 rather than GH3 β-glucosidases have more application advantages in the conversion of soybean isoflavones in the feed industry.

## Electronic supplementary material

Below is the link to the electronic supplementary material.

Supplementary material 1 (DOCX 15 kb)
