# Supplementary material for: A highly glucose-tolerant GH1 β-glucosidase with greater conversion rate of soybean isoflavones in monogastric animals
Source: J Ind Microbiol Biotechnol. 2018 May 9;45(6):369–78. doi: 10.1007/s10295-018-2040-6 (PMC6028883; doi:10.1007/s10295-018-2040-6)
Supplement: Supplementary file 1 — Supplementary material 1 (DOCX 15 kb) [file 10295_2018_2040_MOESM1_ESM.docx]

| **Table 3** Glucose influence on hydrolysis efficiency with exogenous enzyme | | | | | | |
| --- | --- | --- | --- | --- | --- | --- |
|  | GH 1  *As*BG1 | | | GH 3 Bgl3A | | |
|  | Daidzein  (mg/L) | Glycitein  (mg/L) | Genistein  (mg/L) | Daidzein  (mg/L) | Glycitein  (mg/L) | Genistein  (mg/L) |
| Blank | 21.53±1.00 | 3.53±0.31 | 24.04±0.63 | 20.82±0.84 | 3.24±0.61 | 23.42±0.46 |
| Adding Enzyme | 79.21±3.70 | 12.03±1.04 | 118.44±3.11 | 38.14±1.84 | 3.57±0.34 | 37.87±1.02 |
| Adding Enzyme and Glucose | 71.24±3.32 | 10.34±0.90 | 104.47±2.74 | 9.45±0.46 | 2.25±0.21 | 11.63±0.31 |
|  | | | | | | |
| **Table 4** Glucose influence on hydrolysis efficiency without exogenous enzyme | | | | | | |
|  | pH 6.5 | | | pH 4.5 | | |
|  | Daidzein  (mg/L) | Glycitein  (mg/L) | Genistein  (mg/L) | Daidzein  (mg/L) | Glycitein  (mg/L) | Genistein  (mg/L) |
| Control | 4.90±0.20 | 1.16±0.06 | 5.69±0.13 | 5.66±0.18 | 1.75±0.08 | 6.30±0.13 |
| Adding Buffer | 20.35±0.83 | 3.79±0.20 | 23.70±0.53 | 22.35±0.79 | 4.14±0.14 | 24.92±0.50 |
| Adding Buffer and Glucose | 13.59±0.56 | 2.59±0.14 | 16.96±0.38 | 14.86±0.53 | 3.36±0.12 | 18.33±0.37 |

**S. 1. Hydrolysis efficiency of *As*BG1 (GH1) and Bgl3A (GH3) on soy isoflavones in soybean meal**
